# The Comparison of MTT and CVS Assays for the Assessment of Anticancer Agent Interactions

**DOI:** 10.1371/journal.pone.0155772

**Published:** 2016-05-19

**Authors:** Lidia Śliwka, Katarzyna Wiktorska, Piotr Suchocki, Małgorzata Milczarek, Szymon Mielczarek, Katarzyna Lubelska, Tomasz Cierpiał, Piotr Łyżwa, Piotr Kiełbasiński, Anna Jaromin, Anna Flis, Zdzisław Chilmonczyk

**Affiliations:** 1 Department of Cell Biology, National Medicines Institute, Warszawa, Poland; 2 Department of Bioanalysis and Analysis of Drugs, Medical University of Warsaw, Warszawa, Poland; 3 Department of Pharmaceutical Chemistry, National Medicines Institute, Warszawa, Poland; 4 Chair and Department of Synthesis and Chemical Technology of Pharmaceutical Substances, Medical University of Lublin, Lublin, Poland; 5 First Faculty of Medicine, Medical University of Warsaw, Warszawa, Poland; 6 Department of Heteroorganic Chemistry, Centre of Molecular and Macromolecular Studies, Polish Academy of Sciences, Łódź, Poland; 7 Department of Lipids and Liposomes, Faculty of Biotechnology, University of Wroclaw, Wrocław, Poland; 8 Institute of Nursing and Health Sciences, University of Rzeszów, Rzeszów, Poland; Instituto Nacional de Cardiologia, MEXICO

## Abstract

Multiple in vitro tests are widely applied to assess the anticancer activity of new compounds, including their combinations and interactions with other drugs. The MTT (3-(4,5-dimethylthiazol-2-yl)-2,5-diphenyl tetrazolium bromide) assay is one of the most commonly used assays to assess the efficacy and interactions of anticancer agents. However, it can be significantly influenced by compounds that modify cell metabolism and reaction conditions. Therefore, several assays are sometimes used to screen for potential anticancer drugs. However, the majority of drug interactions are evaluated only with this single method. The aim of our studies was to verify whether the choice of an assay has an impact on determining the type of interaction and to identify the source of discrepancies. We compared the accuracy of MTT and CVS (crystal violet staining) assays in the interaction of two compounds characterized by similar anticancer activity: isothiocyanates (ITCs) and Selol. Confocal microscopy studies were carried out to assess the influence of these compounds on the reactive oxygen species (ROS) level, mitochondrial membrane potential, dead-to-live cell ratio and MTT-tetrazolium salt reduction rate. The MTT assay was less reliable than CVS. The MTT test of Selol and 2-oxoheptyl ITC, which affected the ROS level and MTT reduction rate, gave false negative (2-oxoheptyl ITC) or false positive (Selol) results. As a consequence, the MTT assay identified an antagonistic interaction between Selol and ITC, while the metabolism-independent CVS test identified an additive or synergistic interaction. In this paper, we show for the first time that the test assay may change the interpretation of the compound interaction. Therefore, the test method should be chosen with caution, considering the mechanism of action of the compound.

## Introduction

Due to the unsatisfactory effectiveness of existing cancer therapies, new compounds with potential anticancer activity are continuously synthesized. Attempts are ongoing to simultaneously administer a combination of several compounds, which is expected to boost potentiation due to advantageous drug-drug interactions [1].

To screen for potential anticancer compounds and combinations of compounds, multiple assays that measure the effect of the compound on cancer 2D cell culture or tissue-mimicking 3D spheroids are used in preclinical models (in vitro) [2–5]. The compound’s anticancer activity in 2D cell culture is measured using standard indirect and direct assays that determine specific cell culture parameters such as the ability of the cell to proliferate (BrdU staining), the number of dead cells (PI staining), and the number of living cells (cell viability). Indirect tests to determine cell viability such as MTT (3-(4,5-dimethylthiazol-2-yl)-2,5-diphenyl tetrazolium bromide) or CellTiter-Glo utilize the ability of living cells to catalyse reactions, yielding measurable product [6]. The quantity of the product is proportional to the number of living cells. Direct methods include CVS (crystal violet staining), which measures the DNA mass of living cells. In 3D cancer models, these tests have certain limitations. For example, imaging techniques are applied as endpoint readouts. Because these techniques are not compatible with high-throughput screening (HTS) [4,5], 2D cell cultures are most widely used in drug screening and discovery, despite their limitations in mimicking in vivo conditions.

The MTT assay is one of the most popular tests to assess the activity of potential anticancer compounds, and it is also the most popular assay for examining compound interactions. It was created and first described by Mosmann in 1983 [7]. The assay is based on the assumption that MTT tetrazolium salt reduction to formazan occurs in the mitochondria of living cells due to the activity of mitochondrial dehydrogenases (in particular, succinate dehydrogenase). However, the accuracy of the assay has been debated throughout the years. The MTT assay is significantly influenced by compounds that modify cell metabolism by increasing the NADPH level or the activity of LDH *(lactate dehydrogenase)* [8–11]. Maioli et al. showed that rottlerin, which uncouples the mitochondrial respiratory chain, may enhance the production of formazan crystals, leading to false negative results in cell viability assays. Furthermore, MTT tetrazolium salt may be reduced not only in the mitochondria but also within the cytoplasm, on the surface of cell, endosome or lysosome membranes, or even in the extracellular environment [8–12]. Factors influencing the reduction process include the current phase of growth, the cell cycle phase, and reaction conditions such as pH and D-glucose concentration [8,10,12,13].

The CVS assay lacks the limitations undermining the accuracy of MTT and other assays based on enzymatic reactions. It is a simple, non-enzymatic assay for the quick analysis of the quantity of viable adherent cells and colonies [13,14]. The assay takes advantage of the affinity between the dye and the external surface of the DNA double helix. The amount of dye absorbed depends on the total DNA content in the culture and permits the estimation of the number of viable cells in the culture.

Due to the possible shortcomings, using a single assay is associated with the risk of erroneous interpretation. Many papers describe the differences between cell viability/proliferation and cytotoxicity test methods (e.g., XTT, MTT, Alamar blue, Trypan blue, and CellTiter-Glo [15]. Currently, several methods are used simultaneously to examine the anticancer activity of a compound [16]. For compound combinations, only recently [17] have attempts been made to discuss the advantages and disadvantages of data analysis methods used to evaluate the nature of drug-drug interactions. However, it is also crucial to determine if the choice of an assessment method can influence the results of the drug-drug interaction evaluation.

The aims of our studies were to verify whether the choice of a cell viability assessment method has an impact on the determination of the type of interaction and to identify the source of discrepancies.

We chose for our studies, the compounds which have been extensively studied in our laboratory: the anticancer phytocompounds—isothiocyanates (ITCs) which are low-molecular- weight compounds that are present in the form of glucosinolates in vegetables of the Brassicaceae family (broccoli, Brussels sprouts) and Selol—an organic compound of selenium +4 which exhibits lower systemic toxicity than sodium selenite [18–25]. ITCs are thoroughly studied as potential dual treatment compounds along with oxaliplatin [26], 5-fluorouracil [25], paclitaxel [27], and selenium compounds [28]. Sulforaphane (SFN), the most commonly examined ITC, is already in clinical research [29]. Both ITCs and Selol exhibit prooxidative activity, induce apoptosis through the mitochondrial pathway and modulate cellular metabolism [18–25]. The similar activity of Selol and ITCs offers hope for enhancing their anticancer activity by combined administration.

The result of the study may be informative to conduct further research on the combinations of compounds that influence the metabolism of a cell or cellular respiratory chain, e.g. phytocompounds, or compounds used in the clinic, such as doxorubicin [30], platinum [31] and selenium [32] compounds.

The results revealed that the choice of assessment method was crucial for the interpretation of the interaction between Selol and ITCs. The interaction results obtained using the MTT assay indicated antagonism, while the results obtained using the CVS assay indicated advantageous additive or synergistic interactions. The MTT assay was not as reliable as CVS because the MTT tetrazolium salt reduction rate was disrupted by compounds that influenced mitochondrial function and elevated the reactive oxygen species (ROS).

## Materials and Methods

### Compounds

A mixture of selenitetriglycerides (Selol) was synthesized at the Department of Bioanalysis and Drug Analysis, Faculty of Pharmacy of Warsaw Medical University [18]. For our studies, we used Selol containing 5% Se (IV) in the form of lecithin micelles. ITCs were synthesized at the Department of Heteroorganic Chemistry, Centre of Molecular and Macromolecular Studies of Polish Academy of Sciences in Łódź. 5-fluorouracil was purchased from Sigma-Aldrich (St. Louis, MO, USA).

### Cell line

The HT-29 (Catalog No. 30–2007) cell line was obtained from American Type Culture Collection (ATCC, Manassas, VA, USA). There were no reports that the HT-29 cell line was misidentified or cross-contaminated [33]. The culture was cultivated in a 5% CO_2_ atmosphere at 37°C in MEM (Minimum Essential Medium, Cytogen, GmbH Bienenweg, Germany), supplemented with 5% FBS (Fetal Bovine Serum, Sigma-Aldrich Corp., St. Louis, MO, USA), 1% L-glutamine (Cytogen, GmbH Bienenweg, Germany), non-essential amino acids and 1% antibiotics: streptomycin, 10 mg/ml; amphotericin B, 25 μg/ml; penicillin, 10,000 U/ml (Sigma-Aldrich Corp., St. Louis, MO, USA).

### Analysis of anticancer compounds and their combinations

After reaching a confluence of 70–80%, HT-29 cells were trypsinized (0.25% trypsin/EDTA solution, Sigma-Aldrich Corp., St. Louis, MO, USA) and plated in 96-well plates (Cytogen, GmbH Bienenweg, Germany) at a density of 8 x 10^4^ cells/ml.

For MTT and CVS assays after cell adhesion, medium containing increasing concentrations of the investigated compounds was added to the cells, which were then further incubated for 24, 48 or 72 hours. Control cells were incubated with media containing DMSO (ITCs) or micelles with sunflower oil (Selol). After incubation, the cells were rinsed twice in PBS, and MTT and CVS assays were performed.

#### MTT assay

For the MTT assay, 50 μl of 0.25 mg/ml MTT-tetrazolium salts (Sigma-Aldrich Corp., St. Louis, MO, USA) in PBS was added to each well. After 3 hours of incubation, the formazan crystals were dissolved by adding 2-propanol. The absorption of the formazan solution was measured using an Infinite M1000PRO Tecan spectrophotometer at a wavelength of 570 nm [7].

#### CVS assay

For the CVS assay, cells were stained with 0.5% crystal violet (Sigma-Aldrich Corp., St. Louis, MO, USA) in 30% ethanol for 10 minutes at room temperature. The cells were lysed in a 1% SDS (sodium dodecyl sulfate) solution. The absorbance of the solution was measured using an Infinite M1000PRO Tecan microplate spectrophotometer at a wavelength of 595 nm [14].

#### IC_50_ calculations

To assess the compounds’ anticancer potency the IC_50_ values (the concentration that inhibited cell viability to 50% of the control) were determined. The IC_50_ values were calculated from the best-fit (R^2^>0.95) of the Hill slope curve to experimental data using nonlinear regression analysis in Graph Pad Prism (Version 5, GraphPad Software, Inc., La Jolla, USA), according to the formula: Y = 100/1+10^((LogIC_50_-X)*HillSlope)) where X = log of dose, Y = growth inhibition value normalized to control, and HillSlope = unitless slope factor or Hill slope.

#### Interaction assessment

Compound interactions were examined according to the Chou-Talalay method using a multiple drug effect equation [34]. The type of interaction was determined using the combination index (CI) value. The CI method is a mathematical and quantitative evaluation of a two-drug pharmacologic interaction. CI < 1 indicated synergism (the smaller the value, the greater the degree of synergy), CI = 1 indicated an additive effect, and CI > 1 indicated antagonism.

The CI values were calculated using CalcuSyn ver. 2.1 software (Biosoft, Cambridge, UK) according to the formula for mutually exclusive drugs [34]: CI = (D)1/(Dx)1+(D)2/(Dx)2, where (Dx) 1, (Dx) 2 = the concentration of the tested substance 1 and the tested substance 2 used in the single treatment that was required to decrease the cell number by x% and (D) 1, (D) 2 = the concentration of the tested substance 1 in combination with the concentration of the tested substance 2 that together decreased the cell number by x%.

### Mechanism study—microscopic examination

#### Determination of dead to live cell ratio (FDA/PI)

The FDA/PI dye combination discriminates between living and dead cells. Living cells convert FDA to fluorescent fluorescein and exhibit green fluorescence, while dead cells are stained by a membrane impermeable PI and exhibit red fluorescence. This staining and visualization protocol ensured the elimination of the previously described limitations [35]. HT-29 cells were stained with freshly prepared solutions of 0.125 μg/ml FDA and 0.5 μg/ml PI for 15 minutes (Sigma-Aldrich Corp., St. Louis, MO, USA). Then, the samples were analysed using an Olympus IX70 FV500 confocal microscope with a 10x UPlanApo lens. Fluorescence was recorded in the sequential mode to eliminate potential fluorescence bleed-through. The FDA and PI fluorescence excited by an Ar laser with a wavelength of 488 nm and a He-Ne laser with a wavelength of 543 nm, respectively, was collected by 505–525 nm (for FDA) and 560–610 nm (for PI) BP filters [35].

Quantitative data analysis was performed using Fluoview500 software (version 5.0), Olympus, Shinjuk, Tokyo, Japan) and ImageJ (Sun Microsystems, Santa Clara, California) as described previously [36].

#### ROS detection

Dihydrorhodamine 123 (DHR123) was used to assess the ROS level. After reacting with ROS, this dye is oxidized to fluorescent rhodamine 123 (R123). A solution of 12.5 μM DHR123 in PBS was added to each well. The cells were incubated for 15 min at 37°C. The hydrogen peroxide (H_2_O_2_) was used as a positive control (15 μM). Cell fluorescence was observed using an argon laser with a wavelength of 488 nm and a 505–525 nm BP filter. Observations were made with an Olympus IX70 FV500 confocal microscope with a 40x UPlanApo lens within one hour. After that time, the fluorescence intensity decreases compared with the initial value. Because DHR123 is a cationic dye and accumulates in mitochondria, it is sensitive to changes in mitochondrial mass. As was suggested by Forkink et al., the mitochondrial membrane potential (∆Ψm) should be assessed in parallel [37].

#### Mitochondrial membrane potential (∆Ψm) measurement

To determine the influence of Selol and ITCs on mitochondrial membrane potential Ψm, lipophilic cation, a mitochondrial activity marker MitoLight dye was used. ∆Ψm was measured according to the manufacturer's protocol with MitoLight (5,5’,6,6’-tetrachloro-1,1’,3,3’-tetraethylbenzimidazolylcarbocyanine) dye, which selectively accumulates in the mitochondria. In functional and polarized mitochondria the dye aggregates and emits red fluorescence (λ_em_ ~ 590 nm), while in depolarized mitochondria, it accumulates in a monomeric form and its fluorescence shifts to shorter wavelengths (λ_em_ ~ 525 nm–green fluorescence). A 488 nm Ar laser and a 543 nm He-Ne laser were used as light sources. Observations were made with Olympus IX70 FV500 confocal microscope with 40x UPlanApo lens. In order to eliminate the possible fluorescence bleed-through fluorescence was recorded in sequential mode in two channels with the use of 505–525 nm and 560–610 nm BP filters.

#### Visualization of mitochondria

Mitochondria were visualized with MitoTracker DeepRed dye (Thermo Fisher Scientific, Waltham, Massachusetts, USA), which stains mitochondria in living cells regardless of mitochondrial membrane potential. The dye solution was added to wells, and plates were incubated for 10 minutes at 37°C. The confocal images were collected with the Olympus IX70 FV500 confocal microscope equipped with an oil 60x UPlanApo lens. A He-Ne 633 nm laser used as a light source, and the fluorescence signal was collected through the 660 nm BA filter.

#### Microscopic analysis of formazan crystals

A 50-μl aliquot of 0.25 mg/ml MTT-tetrazolium salts in PBS was added to cells. After 3 hours of incubation, formazan crystal production was examined with an Olympus IX70 FV500 confocal microscope equipped with a 40x UPlanApo lens. The Ar laser (488 nm) was used as a source of light [38], and observations were made using the transmitted light channel.

### Statistical analysis

Data are presented as the mean value ± standard deviation (S.D.). The study was conducted in at least three independent runs. The statistical analysis was performed with GraphPad Prism 5 (GraphPad Software, Inc, La Jolla, USA) software using an unpaired t-test. p < 0.05 was considered statistically significant.

## Results and Discussion

### MTT and CVS test application leads to discrepancies in examining the interactions between compounds

**Fig 1:**
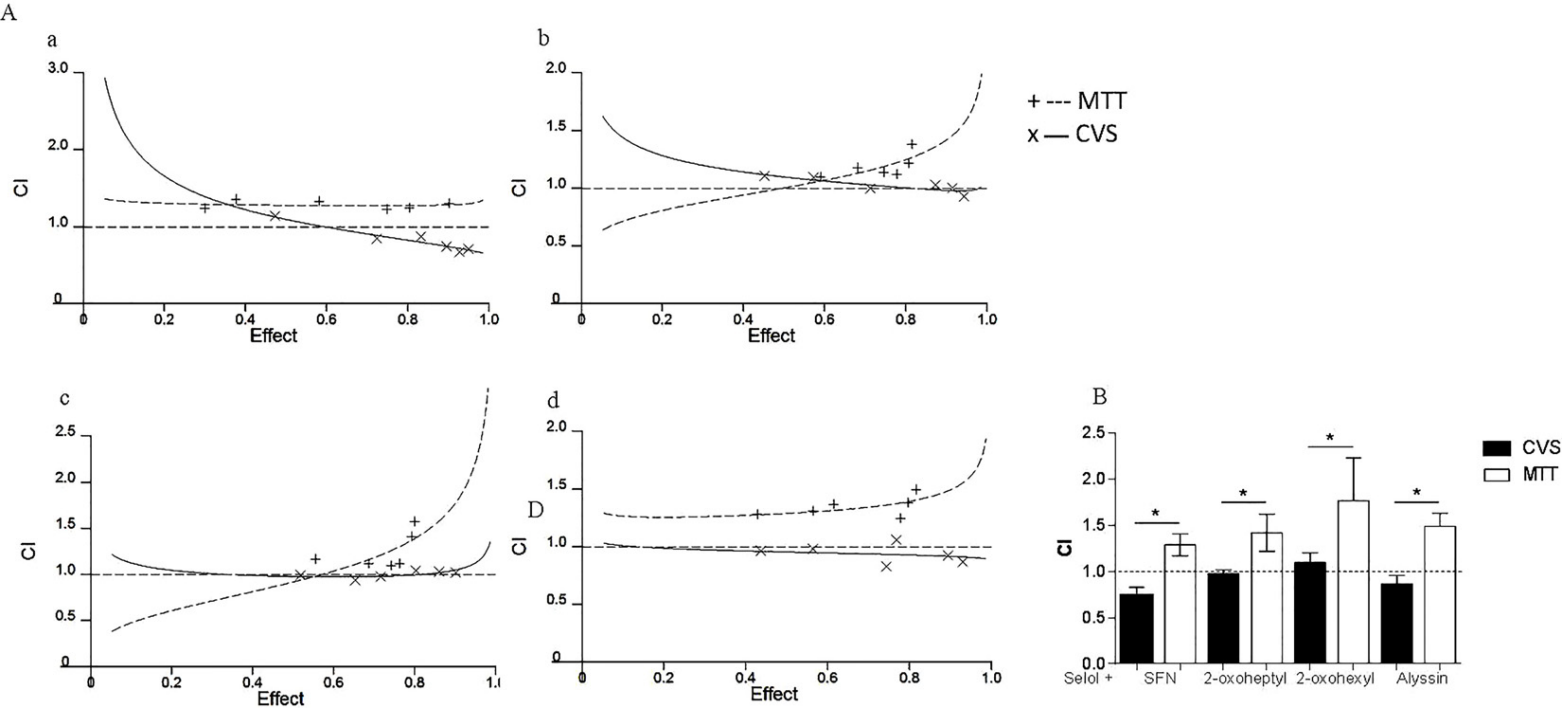
**(A) Effect of the test method on the drug-drug interaction results.** (a) Selol and SFN, (b) Selol and 2-oxoheptyl ITC, (c) Selol and 2-oxohexyl ITC, (d) Selol and alyssin. Combination index (CI) plots present CI as a function of effect (fa = fraction affected, defined as percentage inhibition/100) from 0.05 to 0.95 (5–95% cells killed). CI > 1.0 indicates antagonism, CI = 1 indicates additive effects, and CI < 1.0 indicates synergism. x, experimental data obtained with MTT assay. +, experimental data obtained with CVDE assay. Dashed (for MTT) and solid (for CVS) lines = computer simulation for Fa-CI plot. **(B) Discrepancies between CI calculated on the basis of data obtained with MTT and CVS assays at fa 0.9.** CI > 1.0 indicates antagonism, CI = 1 indicates additive effects, and CI < 1.0 indicates synergism. * significant difference, p < 0.05.

The objects of the interaction study were Selol and ITCs, including SFN and its analogues. SFN and alyssin contain sulfinyl groups in the structure and differ by one–CH_2_ group. 2-oxohexyl ITC and 2–oxoheptyl ITC have theacetyl group instead of the methanesulfinyl moiety and also differ by one -CH_2_ group. To assess the compound interactions, median-effect analysis was applied to calculate the CI from cell viability curves determined by the MTT and CVS methods. In Fig 1A, the CI values for different cell survival ratios (fa = fraction affected) are presented along with the computer simulation for the CI-fa plot. The median-effect plots of single agents and their combinations are included in Supplementary Material (data in S1 Fig).

In the studies of Selol interactions with each investigated ITC using the MTT assay, the CI value increased together with the fa value, which indicated growing antagonism between the compounds. A different pattern of interaction was recorded using CVS. In particular, the results obtained for fa>0.5 were significant in terms of the anticancer activity and showed that depending on the method used, different CI values were recorded for the same combination of compounds. For the combination of Selol with SFN, an increase in fa was accompanied by a decrease in CI until it reached a value of 0.7, indicating synergism. When administering Selol with any of the analogues, the results obtained by CVS indicated an additive nature of the interaction. The differences in the interaction results were most remarkable for fa 0.9 (Fig 1B). The CI calculated based on the CVS assay result was almost twofold lower than that determined using the MTT method.

The results of our research provide essential evidence that the choice of assay method is crucial in the assessment of the interaction type. According to the best knowledge of the authors, the MTT test is the most commonly used assay to assess interactions. As we have shown with Selol and ITCs, use of the MTT assay alone can underestimate the potency of the combined compounds. Exclusive reliance on the MTT assay did not yield data to justify further research on this combination. In contrast, the data obtained using the CVS assay indicate that the combined administration enhanced the activity of Selol and ITC, encouraging further research. The literature features cases of compound combinations that showed an additive interaction in preclinical studies and were eventually used in therapy, including a combination of zoledronate and docetaxel in prostate cancer cells [39] and the combination of trastuzumab and genistein in breast cancer cells [40].

### Differences occur between cell viability results obtained with the MTT and CVS assays

**Fig 2:**
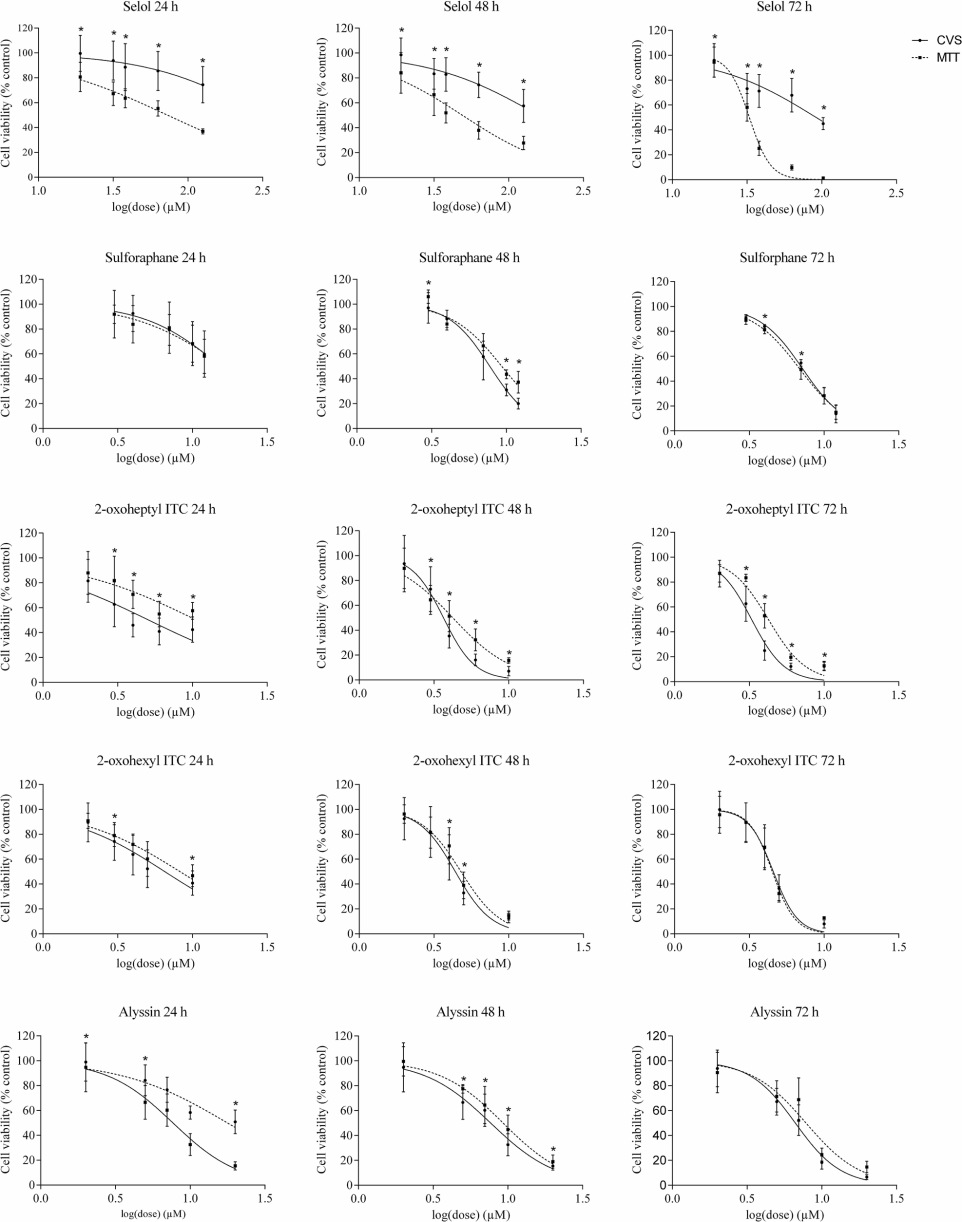
Effect of Selol, SFN, 2-oxoheptyl ITC, 2-oxohexyl ITC and alyssin on cell viability determined by MTT and CVS assays after 24, 48 and 72 h of incubation. Each bar represents the mean ± SD (n = 18). *Statistically significant difference between the growth inhibition effect determined by MTT and CVS assays, p < 0.05.

To determine the accuracy of the test method used to assess Selol and ITC interactions, we performed MTT and CVS tests for each compound studied. The dose-response curves presented in Fig 2 indicate that all compounds decreased HT-29 cell viability. Both the MTT reduction activity and the total DNA mass decreased with compound concentrations and incubation times.

Selol exhibits anticancer properties in human leukaemia HL-60 cells [22], HeLa cervical cancer cells [41] and MCF-7 breast cancer cells [42], whereas the ITC anticancer activity was confirmed in Caco-2 colorectal cancer cells, HT-29, LNCaP prostate cancer cells, HepG2 liver cancer cells [29] and MCF-7 breast cancer cells [43]. As in the present study, these studies reported that the Selol and ITC effect depended on dosage and incubation time.

Among the examined compounds, the most potent was 2-oxoheptyl ITC. The IC_50_ values after 72 hours of incubation were 3.47 μM (CVS) or 4.13 μM (MTT) and were lower than the IC_50_ obtained for the well-established anticancer drug 5-fluorouracil at 6.5 (MTT) and 5.2 (CVS), respectively (Table 1). The least potent ITC was SFN, according to the CVS assay, or alyssin, according to the MTT assay. The IC_50_ calculated for these compounds was 7.18 μM and 7.84 μM, respectively. Published studies have also reported that SFN analogues with acetyl groups were stronger inhibitors of cell growth [21].

**Table 1:** IC_50_ values (μM) of ITCs, Selol and 5-fluorouracil obtained by MTT and CVS assays.

Compound	Sulforaphane			Alyssin			2-oxohexyl ITC			2-oxoheptyl ITC			Selol			5-FU
Time of incubation (h)	24	48	72	24	48	72	24	48	72	24	48	72	24	48	72	72
MTT	16,38± 0,10	9,32± 0,26	6,85± 0,20	17,74± 0,21	9,29± 0,30	7,84± 0,11	8,24± 0,12	4,79± 0,22	4,50± 0,36	10,66± 0,27	4,23± 0,57	4,24± 0,65	103,1± 0,99	65,9± 1,38	32,8± 1,15	6,50± 0,14
CVS	15,41± 0,13	7,75± 0,13	7,18± 0,20	12,63± 0,01	7,67± 0,11	6,65± 0,15	6,52± 0,15	4,39± 0,32	4,57± 0,58	5,11± 0,15	3,66± 0,60	3,27± 0,38	>100	>100	98,6	5,20± 0,32
significance	ns	**	*	*	**	*	*	ns	ns	**	**	***	***	***	***	**

Significance of the differences between methods

* p≤0,05

**p≤0,01

***≤0,001; ns—*non-statistically significant results*

Regardless of the method, Selol proved to be the least effective. The IC_50_ of Selol after 72 hours of incubation was 33.8 μM in the MTT test and over 90 μM when CVS was used to assess cell viability (Table 1). Our findings are complementary to previous observations that Selol exhibits mild cytotoxicity and requires an extended incubation time to inhibit cancer cell growth. Dudkiewicz-Wilczyńska et al. emphasized that this activity of Selol results from the necessity to release selenium from organic bonds (micelles) [41].

Although the order of the compound IC_50_ did not depend on the test method used, the IC_50_ value differed noticeably with respect to the method used. Published studies have also reported differences in the IC_50_ obtained with different tests, including a direct (e.g., cell number count by trypan blue) and indirect assay (MTT) [44, 45].

Analysis of the dose response viability curves showed that the biggest difference between methods was observed for Selol. The drop in cell viability measured by the MTT method was always stronger than that determined by CVS measurements. After 72 hours of treatment with 63 μM of Selol, cell viability dropped to 10% as measured by the MTT method and to 68% as measured by the CVS method.

For SFN and its analogues, the differences between the results obtained using the two methods were not as significant as for Selol Unlike with Selol, the cell viability determined by the MTT assay was higher than that determined by the CVS assay. The largest differences were recorded for the compound with the highest anticancer activity, 2-oxoheptyl ITC. The IC_50_ value obtained with CVS was 1.3 (72 hours)– 2.1 (24 hours) times lower than that obtained by the MTT assay. For Selol, the IC_50_ value was approximately 3 times higher at all time points (Table 1).

It has previously been proposed that the crucial issue in choosing a method to examine the effects of potential anticancer agents is the consideration of the mechanisms of activity of the investigated compounds. Therefore, we conducted a mechanistic study to evaluate which method is more appropriate and what factors are crucial for the accuracy of the method.

### The MTT method is less accurate than CVS

To verify which method better reflects the impact of the studied compounds on the cell culture, the microscopic evaluation of cell number and fractional viability was performed. Microscopic imaging of FDA/PI stained cells discriminated between and counted living and dead cells. As was previously described for adherent cells, microscopy is a more versatile technique, and the dead and live cell number count delivers equal results to cytometry [36].

**Fig 3:**
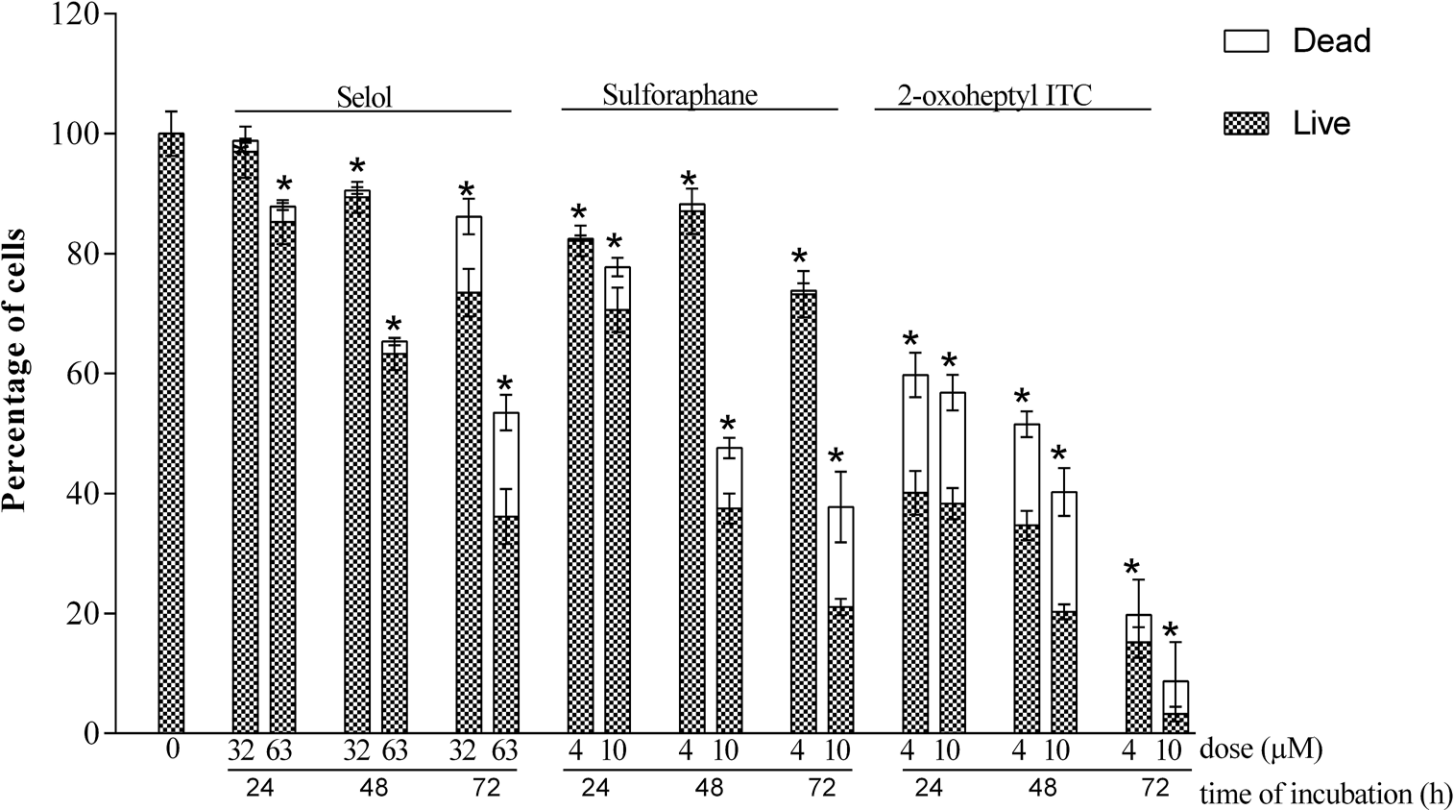
Effects of Selol, SFN and 2-oxoheptyl ITC on total cell numbers and the live/dead cell ratios. Cells were incubated with compounds for 24, 48 and 72 h and stained with FDA/PI. Data analysis was performed using Fluoview500 software (version 5.0) and ImageJ program. * significant difference, p < 0.05.

The MTT and CVS assay results differed the most for Selol and ITC-2-oxoheptyl. As shown in Fig 3, after prolonged incubation with Selol the number of observed cells distinctly decreased, which was accompanied by a decrease in the living cell ratio and an increase in the number of dead cells at 72 hours of incubation. However, these changes were not as substantial as the MTT results had suggested (Fig 1). After 24 hours of incubation, the drop in the living cell number corresponded with the results of the CVS test, while the MTT test yielded lower values of cell viability. After 72 hours of incubation with Selol, especially at a concentration of 63 μM, the MTT test detected almost no living cells in the culture. At the same time, the FDA/PI measurements revealed that cell viability dropped to 50% of the control (Fig 3, data in S2 Fig). Therefore, the MTT method yielded a false positive result for Selol.

For 2-oxoheptyl ITC, a distinct drop in cell number was observed for both 4 and 10 μM, especially after 72 hours of incubation (Fig 3). Dead cells accounting for 20% of the cell population were present only after 24 and 48 hours of incubation (Fig 3). However, a comparison of FDA/PI assay results with the dose-response curves obtained by the MTT and CVS methods (Fig 1) clearly indicates that the CVS method results reflect the actual impact of this compound on cell culture. The ratio of living cells in the culture dropped to 20% with 4 μM after 72 hours of incubation, which was in close proximity with the CVDE test results (24%) but contradictory to the MTT results (54%) (Fig 1).

The MTT and CVS methods gave the same results for SFN. The FDA/PI measurement revealed that the total cell number decreased with incubation time. There were no dead cells at 4 μM, but at 10 μM the number increased with time (Fig 3).

These FDA/PI staining results indicated that the CVS method results better reflected the actual impact of the studied compound on cell culture. The majority of researchers agree that the CVS test recognizes only live cells [14]. However, Chiba postulated that it also counts dead cells and underestimates the cytotoxicity of a compound [13]. Comparing the FDA/PI results with those of CVS indicates that the presence of dead cells does not significantly affect the accuracy of the CVS method. We conclude that the differences between the cell viability results obtained by the CVS and MTT methods exist due to the inaccuracy of the MTT test, which overestimated cell viability in the case of 2-oxoheptyl ITC and underestimated the cell viability in the case of Selol. These differences in the results of MTT and CVS assays yielded the different outcomes of interaction assessment and in consequence the different clinical interpretation of the combination of the studied compounds.

To identify the source of these MTT test discrepancies, we investigated the impact of ITCs and Selol on the mitochondrial status, ROS level and cellular capability to reduce MTT-tetrazolium salt.

### The tested compounds disrupt the MTT-tetrazolium salt reduction rate

#### Selol

**Fig 4:**
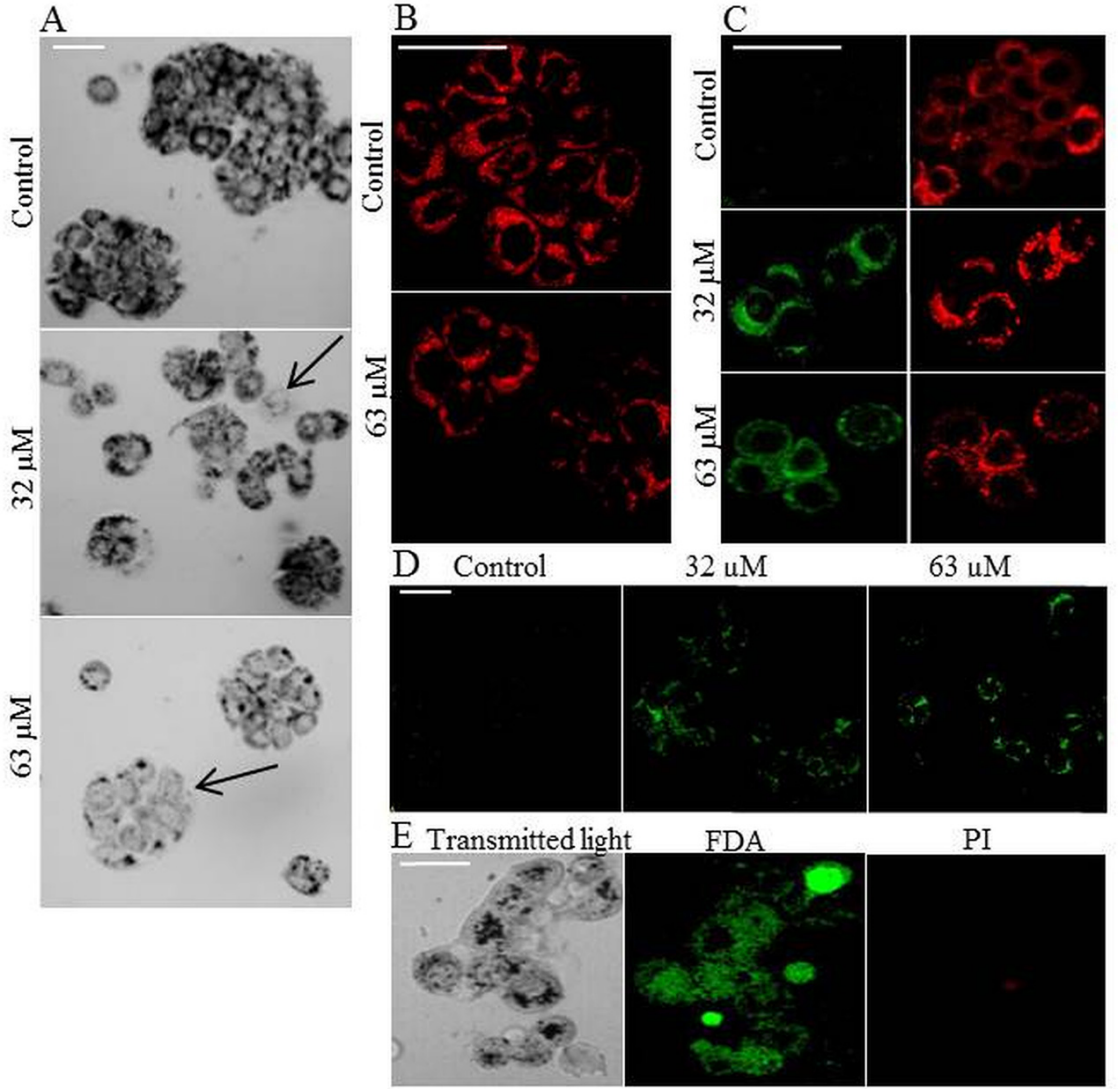
Effect of Selol on MTT reduction. (A) Microscope images of formazan crystals in HT-29 cells. Cells were incubated with 32 and 63 μM Selol for 24 h. Microscope images were recorded after 3 h of reduction of MTT. Black dots indicate formazan crystals. The arrows indicate a cell without formazan. (B) HT-29 cell mitochondria after 24 h incubation with Selol. Cells stained with the MitoTracker® Deep Red. (C) Microscope images of Selol-induced changes in mitochondrial membrane potential (ΔΨm). HT-29 cells were incubated with 32 and 63 μM Selol and stained with MitoLight dye, which stains mitochondria in a membrane potential-dependent fashion. Changes in the mitochondrial membrane potential ΔΨm were detected by confocal microscopy. Left image presents the detection of monomers (green fluorescence), indicating the presence of depolarized mitochondria. Right image presents the fluorescence of the aggregates (red fluorescence), indicating functional, polarized mitochondria. (D) Microscope images of the intracellular reactive oxygen species (ROS) induction by Selol (green fluorescence). HT-29 cells were incubated with 32 and 63 μM Selol and stained with the ROS-sensitive dye DHR123. (E) On the left: Microscope images of formazan crystals in HT-29 cells incubated with 63 μM Selol for 24 h. On the right: FDA/PI staining of HT-29 cells incubated with 63 μM Selol for 24 h. Microscope images were recorded after 3 h of reduction of MTT. Black dots indicate formazan crystals, green fluorescence denotes living cells stained with FDA, and red fluorescence denotes dead cells stained with PI. Scale bar = 50 μm.

In cells incubated with Selol, the quantity of formazan produced varied between cells, as shown in Fig 4A. In some cells, the amount of formazan was similar to control cells, while in other cells it was distinctly lower. Single cells in which almost no formazan crystals were produced were also observed. To determine whether the cells with low numbers of formazan crystals were living or dead, the cells were stained with FDA/PI. As presented in Fig 4B, all of these cells exhibited green fluorescence, indicating that they were all alive. After incubation with Selol, these cells produced undetectable amounts of formazan and were treated as dead in the MTT assay. However, Selol only inhibited the activity of the enzymes engaged in the tetrazolium salt reduction. Berridge previously reported that the MTT assay is not capable of differentiating between dead cells and dormant or inactive cells [12]. This finding explains why the cell viability obtained by MTT assay was significantly lower than that obtained by the CVS assay, which is insensitive to changes in cell metabolic activity.

In the literature regarding the mechanism of activity of both organic and inorganic selenium compounds, it was reported that these compounds disturbed the functions of the mitochondria by inducing oxidative stress [23, 24]. Previous studies on Selol in the non-small-cell lung carcinoma line A549 [32] and leukaemia [22] showed that this selenium compound also induced mitochondrial membrane depolarization.

Our study on the impact of Selol on the mitochondria status and ROS level revealed that in cells incubated with Selol, the mitochondria that are thought to be the place of MTT reduction remain unchanged as observed under a microscope (Fig 4C), while their membranes were depolarized (Fig 4D). Simultaneously, an increase in R123 fluorescence was observed, indicating the elevation of the ROS level (Fig 4E).

A decrease in the mitochondrial membrane potential resulted in the leakage of ROS. When the defence capacity of the cell is overwhelmed, the presence of ROS leads to the disruption of mitochondrial enzyme activity [24]. Scatena et al. showed that under increased ROS level conditions, the activity of mitochondrial succinate, an enzyme responsible for MTT salt reduction, is inhibited [46]. Hence, based on the obtained results we can conclude that the reduced potency of cells incubated with Selol to produce formazan can be assigned to the inhibited activity of MTT-tetrazolium salt reducing mitochondrial enzymes.

#### Isothiocyanates

**Fig 5:**
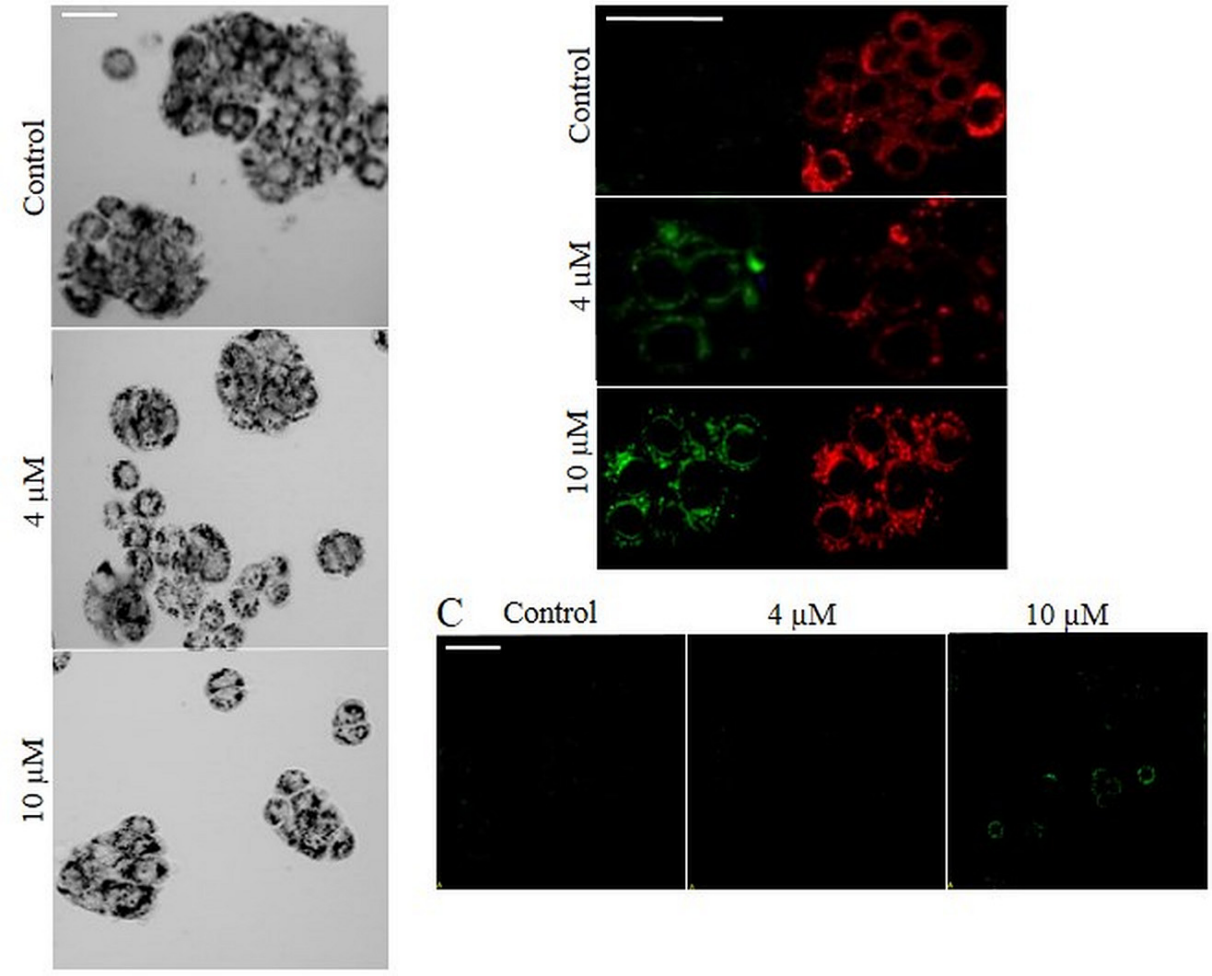
Effect of SFN on MTT reduction. (A) Microscope images of formazan crystals in HT-29 cells. Cells were incubated with 4 and 10 μM of SFN for 24 h. Microscope images were recorded after 3 h of reduction of MTT. Black dots indicate formazan crystals. (B) Microscope images of SFN-induced changes in mitochondrial membrane potential (ΔΨm). HT-29 cells were incubated with 4 and 10 μM SFN and stained with MitoLight dye, which stains mitochondria in a membrane potential-dependent fashion. Changes in the mitochondrial membrane potential ΔΨm were detected by confocal microscopy. Left image presents the detection of the monomers (green fluorescence), indicating the presence of depolarized mitochondria). Right image presents fluorescence of the aggregates (red fluorescence), indicating functional, polarized mitochondria. (C) Microscope images of the intracellular reactive oxygen species induction (ROS) by SFN (green fluorescence). HT-29 cells were incubated with 4 and 10 μM SFN for 24 h and stained with the ROS-sensitive dye DHR123. Scale bar = 50 μm.

Contrary to cells incubated with Selol, larger quantities of formazan were produced in cells incubated with the SFN analogue 2-oxoheptyl ITC than in the control cells (Fig 5A). Formazan was present in two forms: intracellular granules and needle-shaped crystals. The needle-shaped crystals were described previously as exocytosed formazan, which grow only when cells produce a massive amount of formazan [47]. These results indicate that 2-oxoheptyl ITC, unlike Selol, accelerated the rate of MTT-tetrazolium salt reduction.

A similar phenomenon was described for other compounds derived from plants, including EGCG. Increased formazan production resulted from increased succinate dehydrogenase activity [12]. ITCs modulate the activity of cellular enzymes [48]. Therefore, it can be assumed that the increased MTT-tetrazolium salt reduction rate is an effect of elevated succinate dehydrogenase activity. In a comprehensive review of the impact of SFN on mitochondrial functions, Negrette-Guzmán et al. hypothesized that the MTT-salt reduction rate may be accelerated without cell number increase due to the promotion of mitochondrial functions or by induction of mitochondrial biogenesis [49].

As shown in Fig 5, 2-oxoheptyl ITC did not influence the mitochondrial mass. However it induced mitochondrial membrane depolarization and significantly increased the R123 fluorescence. These data indicate that 2-oxoheptyl ITC, like Selol, increased the ROS level, thereby inhibiting mitochondrial succinate activity [47]. Hence, the accelerated formazan production in these cells must be associated with extramitochondrial MTT-tetrazolium salt reduction. Bernas et al. revealed that more than half of formazan production occurs outside of mitochondria [50]. The dehydrogenases and NADP(H) associated with organelles such as the ER [11] or plasma membrane but not the mitochondria are associated with MTT-tetrazolium salt reduction. Reasons for the MTT discrepancies might include extramitochondrial MTT-reducing enzyme activity enhancement in the case of 2-oxoheptyl ITC and mitochondrial enzyme activity inhibition in the case of Selol.

**Fig 6:**
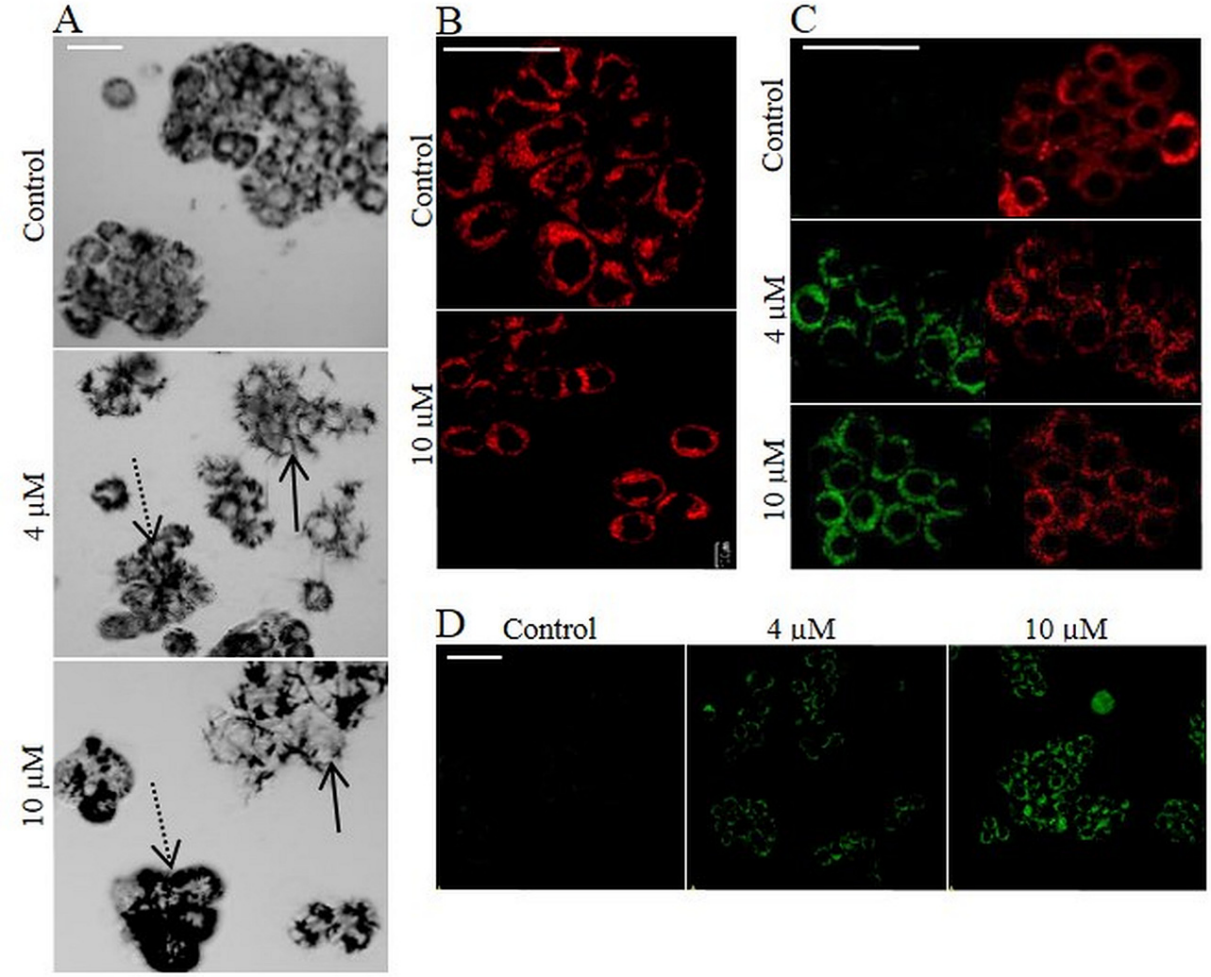
Effect of 2-oxoheptyl ITC on MTT reduction. (A) Microscopic images of formazan crystals. HT-29 cells were incubated with 4 and 10 μM 2-oxoheptyl ITC for 24 h. Microscope images were recorded after 3 h of reduction of MTT. Black dots indicate formazan crystals (dashed line arrow). Needle-shaped formazan crystals larger than the cells are denoted with solid line arrows. (B) Mitochondria of HT-29 cells after 24 h incubation with 2-oxoheptyl ITC. Cells were stained with MitoTracker® Deep Red. (C) HT-29 cells were incubated with 4 and 10 μM 2-oxoheptyl ITC and stained with MitoLight dye, which stains mitochondria in a membrane potential-dependent fashion. Changes in the mitochondrial membrane potential ΔΨm were detected by confocal microscopy. Left image presents the detection of the monomers (green fluorescence), indicating the presence of depolarized mitochondria. Right image presents the fluorescence of the aggregates (red fluorescence), indicating functional, polarized mitochondria. (D) Microscope images of the intracellular reactive oxygen species induction (ROS) by 2-oxoheptyl ITC (green fluorescence). HT-29 cells were incubated with 4 and 10 μM 2-oxoheptyl ITC for 24 and stained with the ROS-sensitive dye DHR123. Scale bar = 50 μm.

As shown in Fig 6A, mitochondrial membrane depolarization was observed for both SFN concentrations (Fig 6B). However, elevated ROS production was only observed at higher concentrations. At lower concentrations, no effect was recorded (Fig 6C). Simultaneously, SFN did not influence the activity of MTT reducing enzymes. The quantity of formazan produced by a single cell incubated with SFN was comparable to that produced by a single control cell. For this compound, the MTT assay results were similar to the CVS assay results.

The microscopic measurements allowed us to explain the grounds of antagonistic character of interaction determined by MTT test method. Selol and ITCs modified the cellular reduction of MTT by deregulating of mitochondrial function and ROS generation, accompanied by the upregulation of mitochondrial dehydrogenases or extramitochondrial MTT reducing enzymes. As a consequence, the antagonistic interaction between Selol and ITC determined by MTT method reflected the effect of the compound combination on the cellular metabolism rather than the cell growth because the opposite effect of Selol and ITCs on the MTT reduction rate was observed.

## Conclusion

This paper has provided new, insight into how and when the choice of the cell growth assessment method may change the outcome and interpretation of the drug-drug interaction study result. We have shown that the unfavourable antagonistic interaction between studied compounds determined by the MTT assay reflected the effect of the compound combination on cellular metabolism rather than cell growth because the opposite effect of Selol and ITCs on the MTT reduction rate was observed. On the contrary, the interaction type determined by the metabolism-independent CVS test was additive or synergistic.

Recently more research is conducted to evaluate the interactions between two or more compounds in the attempt to search for a more effective therapy. There are ongoing investigations of the combinations of cytostatics, interactions of nature-originated phytocompounds with cytostatics as well as well as of the combinations of two phytochemicals [25–28]. Taking into account that both many phytochemicals and cytostatics e.g. doxorubicin or platinium have been shown to influence cellular metabolism or respiratory system [30, 31] the results of this study should be considered while conducting a drug-drug interaction study. Although MTT is the most commonly used method to determine drug-drug interaction type, it may be not reliable. As it was proposed in case of cytotoxicity evaluation of a single compound [13] also in case of the assessment of drug-drug interaction the compounds mechanism of action must be taken into account to choose the appropriate assessment method.

## Supporting Information

S1 Fig Median-effect analysis in HT-29 cells incubated with ITCs and Selol for 72 hours. Column A: data obtained with the MTT assay. Column B: data obtained with the CVS assay. The median effect was calculated using CalcuSyn computer program. ● indicates ITCs (SFN, 2-oxoheptyl ITC, 2-oxohexyl ITC and Alyssin); + indicates Selol, and x indicates Selol plus each ITC. A plot x log (D) versus y log (fa/fu), where fa+ fu = 1 and fu = 1-fa. This plot linearizes all dose-effect curves that followed the mass-action law principle.
(TIF)

S2 Fig Effect of Selol, SFN and 2-oxoheptyl ITC on the live/dead cell ratio in cell culture. Cells were incubated with compounds for 24, 48 and 72 h and stained with FDA/PI. The left image presents living cells stained with FDA, and the right image presents dead cells stained with PI. Scale bar = 100 μm.
(TIF)

S3 Fig Microscopic images of the intracellular reactive oxygen species (ROS) induction by H_2_O_2_. HT-29 cells were incubated with 15 μM H_2_O_2_ for 15 min. and stained with the ROS-sensitive dye DHR123. Left image presents untreated cells, right image presents intracellular reactive oxygen species induction (ROS) by H_2_O_2_ –green fluorescence (Scale bar = 50 μm).
(TIF)
