# Supplementary figures and images for: The Comparison of MTT and CVS Assays for the Assessment of Anticancer Agent Interactions
Source: PLoS One. 2016 May 19;11(5):e0155772. doi: 10.1371/journal.pone.0155772 (PMC4873276; doi:10.1371/journal.pone.0155772)

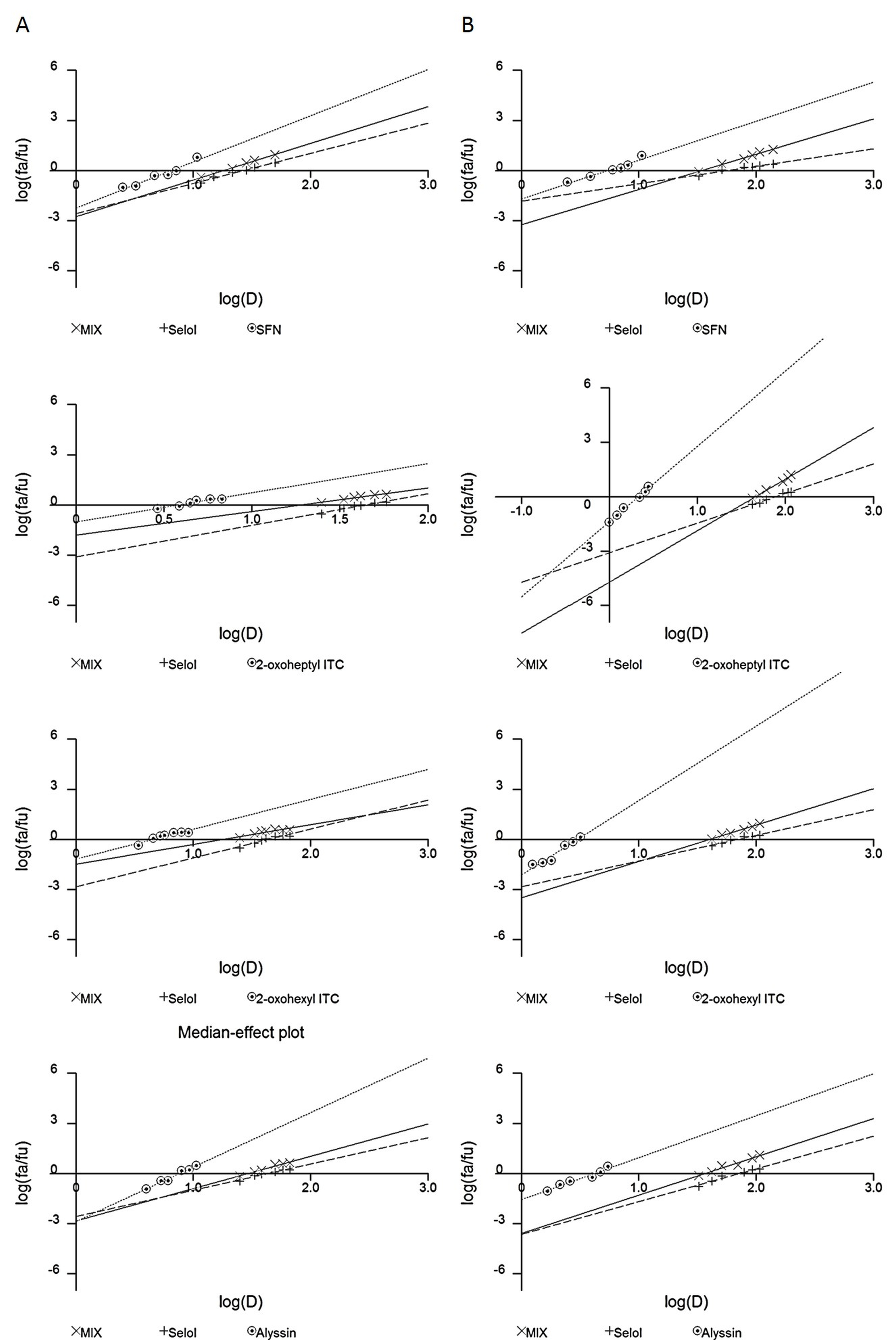

Supplement: S1 Fig — Column A: data obtained with the MTT assay. Column B: data obtained with the CVS assay. The median effect was calculated using CalcuSyn computer program. ● indicates ITCs (SFN, 2-oxoheptyl ITC, 2-oxohexyl ITC and Alyssin); + indicates Selol, and x indicates Selol plus each ITC. A plot x log (D) versus y log (fa/fu), where fa+ fu = 1 and fu = 1-fa. This plot linearizes all dose-effect curves that followed the mass-action law principle. (TIF) [file pone.0155772.s001.tif]

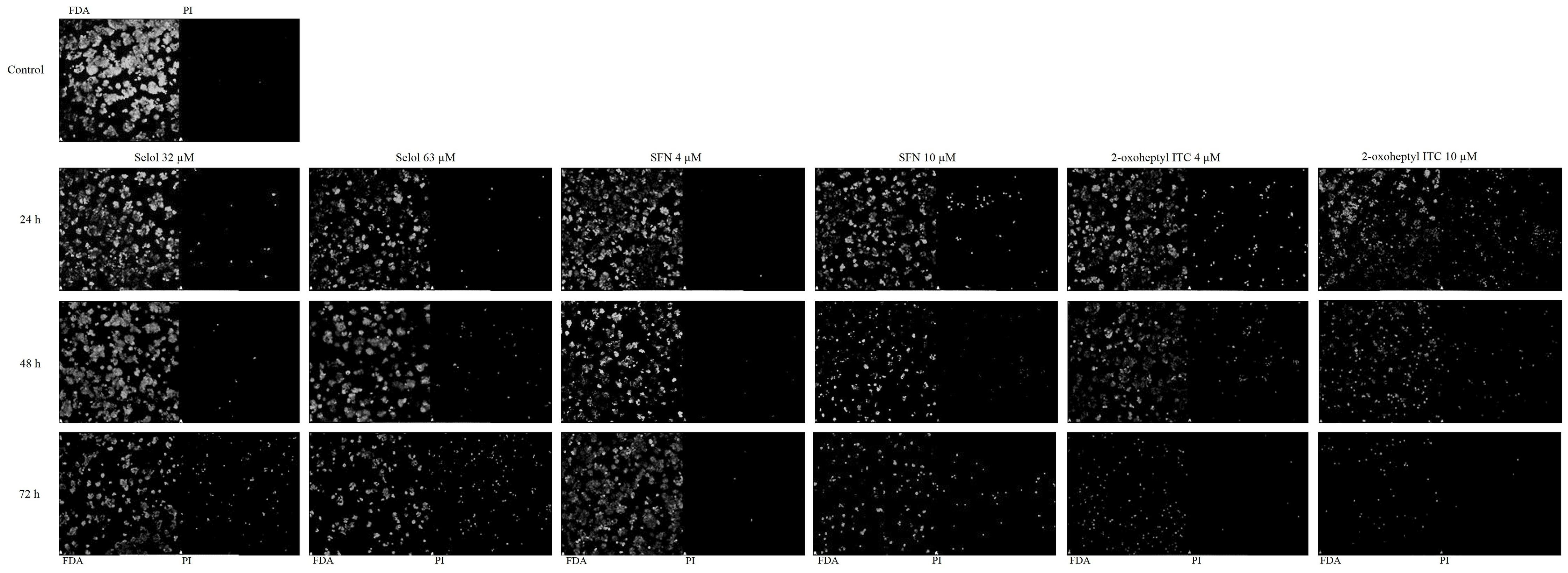

Supplement: S2 Fig — Cells were incubated with compounds for 24, 48 and 72 h and stained with FDA/PI. The left image presents living cells stained with FDA, and the right image presents dead cells stained with PI. Scale bar = 100 μm. (TIF) [file pone.0155772.s002.tif]

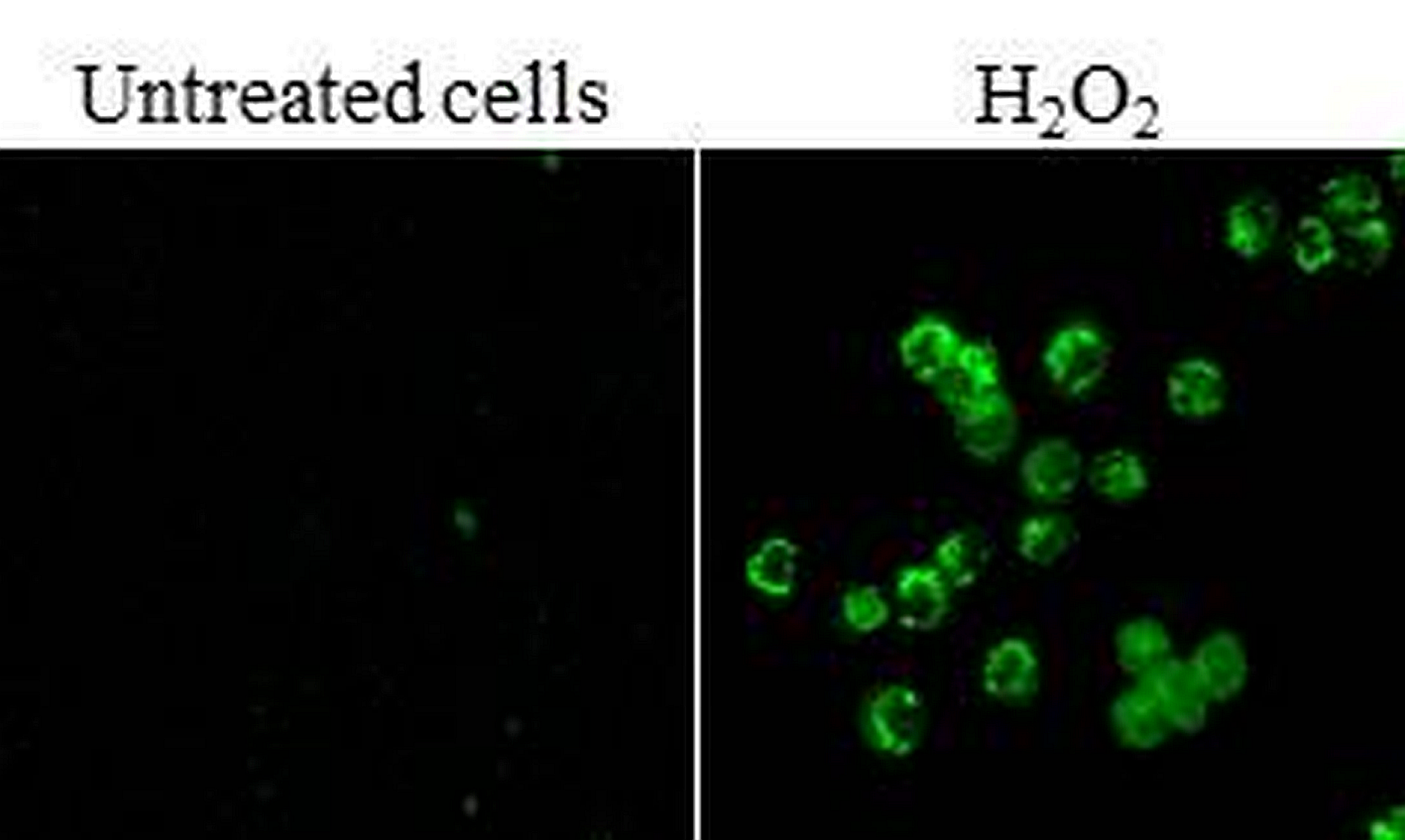

Supplement: S3 Fig — HT-29 cells were incubated with 15 μM H2O2 for 15 min. and stained with the ROS-sensitive dye DHR123. Left image presents untreated cells, right image presents intracellular reactive oxygen species induction (ROS) by H2O2 –green fluorescence (Scale bar = 50 μm). (TIF) [file pone.0155772.s003.tif]
